# Supplementary material for: A multimodal graph neural network framework for cancer molecular subtype classification
Source: BMC Bioinformatics. 2024 Jan 15;25:27. doi: 10.1186/s12859-023-05622-4 (PMC10789042; doi:10.1186/s12859-023-05622-4)
Supplement: Supplementary file 1 — Additional file 1. Detailed Results and Model Settings. [file 12859_2023_5622_MOESM1_ESM.pdf]

Additional file 1: Detailed Results and Model Settings

Table of Results on BRCA Dataset

Table S1: Results of the Proposed and Baseline Models for Cancer Subtype Classification Using the TCGA BRCA Dataset.

Model	300		500		700		1000		2000		5000	
	Accu.[1]	F1	Accu.[1]	F1	Accu.[1]	F1	Accu.[1]	F1	Accu.[1]	F1	Accu.[1]	F1
Proposed w/ GAT	**83.8%**	**0.85**	**84.8%**	**0.85**	**86.4%**	**0.87**	**88.9**	**0.89**	82.8	0.83	81.8	0.82
Proposed w/ GCN	81.8	0.82	81.8%	0.82	83.8%	0.84	84.8%	0.85	**86.9%**	**0.87**	**90.1%**	**0.90**
FC-NN	72.80%	0.73	75.8%	0.76	78.8%	0.79	81.8%	0.80	81.8%	0.80	82.8%	0.81
GCN (Original)	77.8%	0.77	79.8%	0.81	82.8%	0.84	83.8%	0.84	84.8%	0.85	87.9%	0.88
GCN (Modified)	74.2%	0.74	77.4	0.77	81.8%	0.82	81.8%	0.81	78.3	0.78	75.6	0.76
Multi-omics GCN (Original)	77.8%	0.77	79.8%	0.81	81.8%	0.82	82.8%	0.84	84.8%	0.85	86.9%	0.87
Multi-omics GCN (Modified)	81.8%	0.82	82.8%	0.83	82.8%	0.83	83.8%	0.84	84.8%	0.85	85.9%	0.86
GrAMME (Modified)	77.8%	0.77	81.8%	0.82	82.8%	0.84	82.8%	0.84	79.8%	0.79	77.8%	0.79
Multi-omics GAT (Original)	76.8%	0.72	81.8%	0.82	81.8%	0.82	83.8%	0.84	80.8%	0.81	78.8%	0.79
Multi-omics GAT (Modified)	77.8%	0.77	81.8%	0.82	82.8%	0.84	86.9%	0.87	79.8%	0.79	77.8%	0.79

[1] Accu. stands for Accuracy.

Optimal Model Parameters

For the proposed model with the GCN layer, the number of GCN layers used is 2. The output of the dimension-increase layer is 8. The output feature lengths of both the GNN module and the shallow parallel network module are 64. The pooling size is 8. The batch size is 16, and the learning rate is 0.01.

For the proposed model with the GAT layer, the number of GAT layers used is 2. The number of heads is 8. The output of the dimension-increase layer is 8. The output feature lengths of both the GNN module and the shallow parallel network module are 64. The pooling size is 8. The batch size is 16, and the learning
